# Incremental treatment costs for HIV-infected women initiating antiretroviral therapy during pregnancy: A 24-month micro-costing cohort study for a maternal and child health clinic in Kenya

**DOI:** 10.1371/journal.pone.0200199

**Published:** 2018-08-10

**Authors:** Bruce A. Larson, Margaret Bii, Nafisa Halim, Julia K. Rohr, William Sugut, Fredrick Sawe

**Affiliations:** 1 Department of Global Health, Boston University School of Public Health, Boston, MA, United States of America; 2 Kenya Medical Research Institute/Walter Reed Project, Kericho, Kenya; 3 HJF Medical Research International, Inc., Nairobi, Kenya; 4 Harvard Center for Population and Development Studies, Cambridge, MA, United States of America; The University of Warwick, UNITED KINGDOM

## Abstract

**Background:**

To date, little information exists on the costs of providing antiretroviral therapy (ART) within maternal and child health (MCH) clinics in Kenya. The main objective of this analysis was to estimate the annual incremental cost of providing ART within a MCH clinic for adult women initiated on ART during pregnancy over the first one and two years on treatment. The study site was the District Hospital in Kericho, Kenya.

**Methods:**

A micro-costing approach from the provider’s perspective, based on a retrospective review of patient medical records, was used to evaluate incremental costs of care (2012 USD). Cost per patient in two cohorts were evaluated: the MCH clinic group comprised of adult women who initiated ART at the site’s MCH clinic during pregnancy between 2008–2011; and for comparison, the ART clinic group comprised of adult, non-pregnant women who initiated ART at the site’s ART clinic during 2008–2011. The two groups were matched on age and baseline CD4 count at initiation. Retention at year one/two on ART was defined as having completed a clinic visit at 365/730 days on ART +/- 90 days.

**Results:**

For patients defined as retained in care at year one, average incremental costs per patient were $234 for the MCH clinic group (median: 215; IQR: 186, 282) and $292 in the ART clinic group (median: 227; IQR: 178, 357). ARV and laboratory costs were less on average for the MCH clinic group compared to the ART clinic group (due to lower cost regimens and fewer tests), while personnel costs were higher for the MCH clinic group.

**Conclusions:**

The annual incremental cost per patient of providing ART were similar in the two clinic settings in 2012. With shifts in recommended ARV regimens and lab monitoring over time, annual costs of care (using 2016 USD unit costs) have remained relatively constant in nominal terms for the MCH clinic group but have fallen substantially for the ART clinic group (from nominal $292 in 2012 to nominal $227 in 2016).

## Introduction

Kenya remains a country with a substantial burden of disease from HIV. In 2015, an estimated 1.5 million Kenyans were living with HIV (5.5% of men and 6.3% of women), with 77,000 new infections and 35,000 deaths from AIDS.[1] While mother-to-child transmission (MTCT) rates fell from 14% in 2013 to 8.3% in 2015, only 7 of 47 countries had achieved the 2019 target of 5% or less MTCT.[1] Globally, 90% of all HIV-infected pregnant women live in just 22 countries, one of which is Kenya, and an estimated 12% of pregnancy-related deaths and 4.9% of child deaths (< 5 years of age) globally are attributable to AIDS.[2]

Kenyan guidelines for the use of antiretroviral therapy (ART) for pregnant and postpartum women, as part of the overall package of prevention-of-mother-to-child-transmission of HIV/AIDS (PMTCT), have evolved substantially over the past decade. In the 2009, 2011, and 2012 national guidelines, the basic criterion for initiating life-long triple therapy during pregnancy was CD4 < 350 cells/mm^3^_._[3–5] While a higher CD4 eligibility threshold was included in the 2014 guidelines (CD4 < 500 cells/mm^3^), life-long ART for pregnant women (Option B+) was considered preferable to ART only for a limited time after delivery (Option B) for those not otherwise eligible based on CD4 count or WHO stage.[6] As of 2016, all HIV-infected individuals in Kenya are eligible for ART regardless of CD4 count or WHO stage.[7]

While treatment guidelines have been very explicit on when to start ART, and which regimens are preferred, the “where to initiate” ART for pregnant women was initially less clearly identified in guidelines. Beginning with the 2011 guidelines, however, the location of initiation was addressed as “in settings with the capacity to initiate and monitor triple therapy on HIV-infected pregnant women, triple ARV prophylaxis can be used” (see page 152 in [4]). The same idea, which effectively suggested that maternal and child health (MCH) clinics initiate and manage all pregnant and post-partum women on ART, has been incorporated into Kenyan policy through the 2016 revised guidelines where same day initiation after HIV testing at a first antenatal care visit is recommended (see page 108 in [7]).

Integrating ART for pregnant and post-partum women into the services provided in MCH clinics is a logical idea to support initiation of treatment and retention during and after pregnancy.[8] Experience suggests that pregnant and post-partum women face barriers seeking HIV/AIDS care and treatment, including transportation costs, dislike of a more general HIV/AIDS care and ART clinic, and stigma [9–13], so providing ART as part of ante- and post-natal care (ANC/PNC) within MCH settings seems logical. A 2013 literature review, based on fairly limited data, concluded that initiation and provision of ART in MCH clinics improved enrollment on ART among pregnant and post-partum women but rates of retention were similar to un-integrated, referral-based models.[8]

While the logic of integrating ART into MCH services is clear, to date there has been little analysis of the costs of providing ART in MCH settings in general and none in Kenya. For example, a 2011 literature review included only a few studies of “PMTCT unit costs” that mainly focused on single-dose Nevirapine around delivery.[14] More recently, detailed patient-level data from MCH clinics in Zambia were used to estimate the costs of PMTCT service delivery from the first ANC visit through 6 months post-partum, but the costs of ART were not included in the definition of PMTCT service delivery.[15]

The primary objective of this paper is to estimate the annual incremental cost of providing ART within an MCH clinic for adult women initiated on ART during pregnancy over the first one and two years on treatment. The study site began to initiate ART for HIV-infected pregnant women in 2005. For perspective, these costs are also compared to costs over the same time period for a cohort of adult, non-pregnant women who initiated ART at the general ART clinic at the same hospital compound.

This comparison group should not be interpreted as a control group. However, the results for the two study groups provides additional context for understanding and interpreting key drivers of the costs of ART over time in the different clinic settings. Given policy changes over time in Kenya, it was not logical or ethical to randomize pregnant women to initiate ART either at an MCH clinic or a general ART clinic.

## Methods

### Overview

The study site was the Kericho District Hospital (KDH), which has since been renamed as the Kericho County Referral Hospital. The hospital’s physical compound includes multiple buildings. One building is dedicated to the MCH clinic, which provides antenatal care for pregnant women as well as postnatal care for women and their infants (e.g., growth monitoring, vaccinations). The MCH clinic also provides all PMTCT services, which include ART for pregnant women through 18 months post partum (or the cessation of breastfeeding). Another building houses the general ART clinic, which provides HIV care and treatment services to adults, children and adolescents. The two buildings are a short distance apart.

Data for this analysis come from a retrospective review of outpatient medical records (paper records) for the two cohorts of patients. The MCH clinic group is comprised of 79 adult women (≥ 18 years) who initiated ART at the KDH MCH clinic during pregnancy during 2008–2011, who had a medical record available at the time of enrollment for retrospective records review, and who did not formally transfer to another clinic for care during the first year on ART. During this period, the basic life-long ART eligibility criterion remained CD4 < 350 cells/mm3 or WHO Stage III/IV.

The ART clinic group (n = 79) is comprised of adult, non-pregnant women (≥ 18 years) who initiated ART at the ART clinic during same study period and who had a medical record available for review at the time of enrollment. Women included in the ART clinic group were generally matched to women in the MCH group based on age (+/- 5 years) and baseline CD4 count (+- 50 cells/mm3 at ART initiation). For reference, the average age was 31.2/29.9 years and the average CD4 cell count at initiation was 182/174 cells/mm^3^ in the MCH/ART clinic groups respectively.

A micro-costing analysis from the provider’s perspective, based on detailed information for each patient at each visit, is used in this analysis following methods applied in other cost-outcomes analyses of ART completed in Kenya, South Africa, and Zambia.[15–21] Patient-level data on resource utilization were extracted retrospectively onto clinical record forms from outpatient medical records for each visit for each patient at their clinic for at least 24 months after initiation of ART. Data from the clinical record forms were then entered into a study-developed database (CSPRO), with double data entry for quality control purposes. Data were then exported to STATA for further data management and the creation of additional variables needed for the costing analysis. Unit costs (USD 2012) for three key categories of incremental costs–ARV medications, staff encounters, and laboratory tests–were estimated based on financial reports, procurement records, and site records. Costs per patient for these key cost categories were calculated from the provider’s perspective in 2012 Kenyan Shillings (KES), and converted to US dollars (using 84.53KES/$ as the average annual 2012 exchange rate).[22]

### Follow-up period and classification of patient retention outcomes

The follow-up periods for the evaluation of costs is the time period that spans the first and second year on ART. Two main retention outcomes are evaluated: (1) retained in care at one year on ART (365 days), defined as having at least one clinic visit during day 275–455 on ART (365 days +- 90 days); and (2) retained in care at two years on ART (730 days), defined as having at least one clinic visit during day 640–820 on ART (730 days +- 90 days).

Loss to follow-up has been defined in prior studies as being 90 or more days late for a scheduled appointment or drug pick-up (and who are not known to have transferred out or died).[23] A 90-day prescription was typically the maximum supply of ARVs provided during the first year on ART for patients by the study clinics during the study period, so a patient who does not have a visit in the 275–365 day window is lost to follow up at year one, while a patient with a visit during this window is retained in care at year one. This definition of ‘retained at one year’ is also consistent with prior literature.[15–21] By the end of the second year on ART, most women retained on ART at the MCH clinic would have been transferred to the general ART clinic for continued HIV care and treatment.

Patients who are not retained at one year on ART, by definition, had days during the year without ARVs. In addition, patients retained in care at one and/or two years on ART may nonetheless have been late for visits during the year, where late is defined as having days without ARVs between visits (e.g., covered by prescriptions as defined in [24]). For example, a patient might initiate ART, receive a 14-day supply, but then return for their next clinic visit in 20 days. In this case the patient is late, and had 6 days without ARVs. At this second visit, the patient might receive 30 days of ARVs but return after 25 days. At the third visit, the patient still has 5 days of ARVs and might receive another 30-day supply. In this case, the patient is only late if he/she returns after 36 or more days. To highlight this issue in more detail, for patients retained at one year on ART and two years on ART, the total numbers of days without ARVs (days missed) during the year is also reported.

For this analysis, the retention outcomes are best interpreted as retention at the clinic, rather than overall retention in care and treatment. Retention in care has been very difficult to assess correctly using routine medical records in part because patients may switch clinics for care, and information on such transfers may not be well recorded in medical records (e.g., self transfers).[25]

Routine viral load monitoring was not the standard of care during the study follow-up period, and few viral load tests were completed for the two cohorts. As a result, it is not possible to include viral suppression at one and/or two years on ART as a primary or secondary study outcome.

For the evaluation of costs, costs for the first year on ART are based on resources used or provided for patient care between day 0 and day 365 on ART. For example, a patient might have a clinic visit on day 350 on ART, and then receive 90 days of medications. Since the medications are provided at day 350 on ART, the cost for these medications are included in costs for year one on ART. Costs for the second year on ART are based on resources used or provided for patient care between day 366 and day 730 on ART.

### Unit costs

Previously published methods were followed for estimating unit costs for variable resources utilized at the treatment sites by study patients.[15–21] The unit costs of ARV drugs, on a per day basis for each ARV medication dispensed to patients, were estimated as the average unit cost of a particular ARV medication purchased for the PEPFAR South Rift Valley HIV program for use in 2012 (obtained from UNITAID/Clinton Foundation and USAID/Kenya PHARMA). Data on the specific ARV medication prescribed were used (e.g., once-a-day, single pill, fixed-dose triple combination; twice a day, two pills using a double combination plus a single drug; or twice a day, 3 pills with three separate drugs).

Both study clinics use the same laboratories for diagnostic tests, a fee schedule exists for each test, and these fees were used as 2012 unit costs. The study was not designed to evaluate if the fees charged are somehow “correct” or to breakdown these fees into ingredients (equipment, reagents, staff, etc.).

In both study clinics, nurses, clinical officers, and pharmacists are the main categories of health workers providing ART-related care to patients. In addition, based on referral by a clinical officer, a patient might also receive care from a medical doctor, counselor, or other types of specialists. At each visit, information on which types of health care professionals were seen by the patient, called an encounter for short, was abstracted from the patient’s medical file.

Because a market price or fee per staff encounter does not exist at the study sites, a top-down approach was applied to estimate these unit costs. Based on the number of each type of health worker at the clinic, estimates of their time allocated to working in each clinic, and salary information for 2012 (the annual full cost to employer based on government salaries and allowances), the total monthly cost for each category of staff directly providing patient care were estimated. Based on average monthly patient visits to each clinic, and data on which professionals were seen on average at these visits, the number of patient encounters with each type of health worker per month estimated. Monthly labor costs for each health worker category were then divided by the number of patients encounters for each category of health worker to develop a unit cost (cost per encounter) for each category of health worker.[15,16,18,20] Unlike time-motion type analyses that tend to focus on the actual time a health worker spends with a patient, the approach followed here includes any non-patient clinical staff time, such as paper work and breaks, into the cost of patient care.

### Resource use per patient

Based on the detailed patient-level data for each clinic visit during the study follow-up periods (one and two years on ART), the total quantity of each resource utilized (ARV medications, staff, and laboratory tests) for each patient was estimated over the first and second year on ART. The quantity for ARV medications is the number of days of each ARV medication prescribed over the follow-up period. The quantity for staff encounters is the number of encounters with each type of staff over the follow = up period. The quantity for laboratory tests is the number of each type of test ordered over the study follow-up period.

### Incremental costs per patient

Incremental cost for each patient is the unit cost for each resource multiplied by the total quantity utilized over a specific time period. The average incremental cost per cohort over each follow-up period (year one and two) includes costs for patients not retained as well as patients retained in care. Thus, prior literature has reported average costs for the whole cohort, average costs for the subset retained in care and average costs for the subset not retained, as well as the cost to produce one patient retained in care (average costs for the whole cohort divided by the proportion retained in care).[15–21]

For this analysis, the focus is on incremental costs over the first and second year on ART for patients retained at each follow-up period. The goal of HIV care and treatment programs is to have patients retained on ART for life. Therefore, average costs for the subset retained in care provides better information on costs for services clinics intend to provide as well as to understand how costs might evolve with alternative models of care for stable patients (e.g., fewer visits, longer drug prescriptions).

### Modeled incremental costs per patient

Between 2012 and 2016, the recommended first-line drug regimens evolved, with TDF+3TC+EFV becoming the recommended first-line regimen for adults.[7] Between 2012 and 2016, the cost per day of this regimen fell by about 50% (in nominal USD). However, the lower 2016 cost for TDF+3TC+EFV was still about 75% higher in nominal terms than the cost for the common first-line regimens in 2012 (D4T/AZT+3TC+NPV).

In addition, viral load monitoring after ART initiation has largely replaced CD4 monitoring, and the cost for viral load monitoring fell substantially from 2012 to 2016.

Incremental costs for 2016 (USD) are also modeled based on the following assumptions: (1) the same quantities of resources are provided to patients, but TDF+3TC+EFV is used as the first-line regimen; (2) CD4 tests are replaced with viral load tests for patient monitoring; (3) the 2016 unit costs for drugs and laboratory tests are used; and (4) the unit cost for staff encounters in USD are held constant.

The unit cost of staff encounters are held constant in USD for the following reason. Between 2012 and 2016, Kenya experienced 27% inflation in Kenyan shillings (KES), but the exchange rate (KES/USD) fell from 84.5 to 101.5 over the same period.[22] If health worker salaries kept pace with inflation, then encounter costs in 2016 USD would be about 6% high than their 2012 values. If health worker salaries did not keep pace with inflation, then the increase would have been less. In the extreme, if salaries remained constant in KES, encounter costs in 2016 would have been 17% lower in 2016 USD. To focus attention on the two major policy changes over the period (new regimen and viral load monitoring), salary encounter costs are held constant in 2016 USD.

### Ethics

The study was approved by the Scientific and Ethics Research Unit of the Kenya Medical Research Unit (Protocol #2221) and the Institutional Review of the Boston University Medical Center (H-31185). Both IRBs waived informed consent for this retrospective study.

## Results

### Retained in care at 12 and 24 months on ART at the study clinics

For the MCH group (n = 79), 83.5% were retained at one year on ART (n = 66). Four of these patients formally transferred to another clinic during their second year on ART, so n = 75 patients in the MCH group had at least two years of follow-up. For the MCH group with at least two years of follow- up, 76% were retained at two years on ART (n = 57). For perspective, wide variation in retention rates at one year on ART for women initiating ART during pregnancy have been reported, from < 50% to 70–90% depending on the country, context, and cohort details.[26][27].

For the ART clinic group (n = 79), 93.7% were retained at one year (n = 74). No patients in the ART clinic group transferred during year two on ART. For the matched ART clinic cohort with at least 2 years of follow up (n = 75), 82.7% were retained at two years on ART. For reference, retention at one year on ART was 82% at the same ART clinic for a cohort of patients (included both adult men and women) who initiated ART during 2007.[18] Although other estimates of retention at two years on ART for the same ART clinic have not been published, retention was very similar (81%) for a cohort of adults initiating ART during 2010–2012 at a large public sector ART clinic in South Africa.[28] Retention for the ART clinic cohort reported in Table 1 is likely to be better than the clinic average for all adults for at least two reasons related to matching for creation of the ART clinic cohort. First, the ART clinic cohort is all non-pregnant females, and retention of men is typically worse than for women. And, second, the baseline CD4 count for ART eligibility for the matched ART clinic group was likely higher than the ART clinic average because pregnant women tend to have higher baseline CD4 counts at treatment initiation than non-pregnant women.

**Table 1:** Retention outcomes and missed ARV days by clinic.

	MCH clinic	HIV Clinic
Total patients for 1 year analysis	79	79
Retained in care at 1 year	0.835	0.937
Total patients retained at 1 year	66	74
Mean (95% CI) days without ARVs during year 1	53.9 (37.7, 70.0)	28.4 (18.9, 37.9)
Median (IQR) days without ARVs during year 1 if retained	28.5 (12–68)	7 (1–54)
Proportion of days with ARVs > = 90% if retained	0.56	0.72
Total patients for 2 year analysis	75	75
Retained in care at 2 years	0.760	0.827
Total patients retained at 2 years	57	62
Mean (95% CI) days without ARVs during year 2	52.1 (36.2, 67.9)	28.1 (15.7, 40.5)
Median (IQR) days without ARVs during year 1	31 (5–77)	5.5 (0–38)
Proportion of days with ARVs > = 90% if retained	0.54	0.73

Table 1 also reports the mean (95% confidence interval) and median (IQR) for days without ARVs during the first year on ART for the subset defined as retained in care at year one. In the MCH group, the median days missed was 28.5, with 25% of women missing more than 68 days during the year. In the ART clinic group, the median days missed was 7, with 25% of the cohort missing more than 54 days during the year. Using the definition of adequate adherence (≤ 10% of days without ARVs) reported in [24], 56% of patients in the MCH clinic group and 72% of patients in the ART clinic group had adequate adherence.

### Quantity of resources used

Table 2 summarizes the quantities for ARV medications prescribed for year one and two on ART for the subset of patients retained in care at each follow-up period. Quantities for the subset not retained at each endpoint are not reported in Table 2. Patients not retained were prescribed fewer medications, which is almost by definition because they typically stopped coming to the clinic after 3–6 months.

**Table 2:** Average days of ARV medications prescribed during year one and year two on ART (for subset retained in care at each time period).

		Year 1	Year 2	Year 1	Year 2
		MCH clinic	MCH clinic	ART clinic	ART clinic
ARV Medications	Number retained^a^	66	57	74	62
AZT/3TC/NVP (300/150/200 mg)		212.85	178.70	46.93	59.27
D4T/3TC/NVP (30/150/200 mg)		91.79	73.39	137.46	114.58
TDF/3TC/EFV (300/300/600 mg)		0.00	0.00	22.84	20.03
D4T/3TC (30/150 mg)		5.85	10.00	119.47	89.48
AZT/3TC (300/150 mg)		28.45	35.51	58.78	55.65
TDF/3TC (300/300 mg)		6.58	25.19	0.00	0.97
NVP (200 mg)		6.58	23.61	0.00	0.97
EFV (600 mg)		27.09	38.14	165.69	132.55
LPV/r (200/50 mg)		6.09	6.84	0.00	0.00
AZT (300 mg)		0.97	0.00	0.00	0.00
ABC/3TC (300/150 mg)		0.00	0.53	0.00	0.00
3TC (150 mg)		0.00	0.00	0.41	0.00
Total days of ARVs^b^		345.52	322.79	362.65	319.95

^a^The numbers retained are the number retained at one year and two years on ART respectively (from Table 1).

^b^Defined as the sum of days of 3-drug and 2-drug combinations.

Given the number of possible pill combinations prescribed, the last row of Table 2 estimates the total number of days of any triple ARV regimen prescribed over each year by adding up all single pill triple drug combinations as well as all two pill combinations (2 drugs in one pill and the third drug in a second pill).

The average number of laboratory tests performed for the subset of patient retained at year one and year two on ART are provided in Table 3. Five types of laboratory tests were commonly completed (average of 1.21–1.55 tests per patient per year): a full hemogram, CD4 count assay, kidney (creatinine) and liver function tests (alanine aminotransferase (ALT), and aspartate aminotransferase (AST). The quantities of tests changed little between year one and year two on ART, and there was little difference in quantities between the two study groups.

**Table 3:** Average annual number of tests performed if retained in care at one and two years on ART by study group.

		Year 1	Year 2	Year 1	Year 2
		MCH clinic	MCH clinic	ART clinic	ART clinic
Laboratory test	Number retained	66	57	74	62
Full hemogram		1.38	1.42	1.55	1.21
CD4 Count Assay		1.26	1.46	1.49	1.29
Alanine Aminotransferase (ALT)		1.23	1.23	1.49	1.23
Ceatinine		1.20	1.21	1.43	1.19
Aspartate Aminotransferase (AST)		1.17	1.19	1.39	1.16
Venereal Disease Research Laboratory test (VDRL)		0.33	0.44	0.30	0.31
Pregnancy		0.08	0.09	0.08	0.11
Sputum Acid Fast Bacilli		0.03	0.04	0.05	0.02
Hepatitis		0.14	0.14	0.18	0.13
Electrolytes		0.03	0.12	0.05	0.15
HIV Viral Load		0.00	0.05	0.64	0.31
Chest Xray		0.02	0.00	0.01	0.02
Glucose		0.05	0.00	0.03	0.00
Blood Spears (BS) Malaria		0.00	0.04	0.03	0.02
Erythrocyte Sedimentation Rate (ESR)		0.02	0.02	0.00	0.00
Cervical		0.00	0.00	0.00	0.00
Stool for ova, cysts and parasite (OCP)		0.00	0.00	0.00	0.03
Widal		0.00	0.04	0.03	0.05
Histology		0.00	0.00	0.01	0.00
Pelvic		0.00	0.00	0.01	0.00
Bone Marrow (BM) Aspiration		0.00	0.00	0.01	0.00
Urinanalys		0.00	0.00	0.22	0.02
XRAY		0.00	0.00	0.00	0.00
Prothrombin time (PT) test (INR)		0.00	0.00	0.00	0.05
CT Scan		0.00	0.00	0.00	0.02
Ultrasound		0.00	0.00	0.00	0.00
Resistance		0.00	0.00	0.01	0.00

Viral load tests were somewhat common for the ART clinic group during the first year on treatment (on average less than one test in the first year). Routine viral load monitoring is now included in Kenyan treatment guidelines at 6 and 12 months and then annually [7] for all adults, but routine viral load monitoring was not yet fully integrated into treatment guidelines during the full study period. Beyond the seven types of tests listed above, the remaining tests listed in Table 2 were ordered very infrequently.

The average number of encounters with clinic staff for the subset of patient retained at year one and year two on ART are reported in Table 4. In the study sites, as is common in Kenya, patients primarily receive care from clinical officers, nurses, and pharmacists, and generally but not always see each of these types of staff at each visit. Interactions with other staff categories were substantially less common for both groups. For patients retained at year 1, they made on average 10 visits to the MCH clinic (coincides with the number of encounters with a nurse and clinical officer), falling to about 7 visit in the second year (for those retained). These numbers were somewhat lower for the ART clinic group (8 and 5 respectively).

**Table 4:** Average number of staff encounters per patient retained in care at one/two years on ART.

		Year 1	Year 2	Year 1	Year 2
		MCH clinic	MCH clinic	ART clinic	ART clinic
Staff category	Number retained	66	57	74	62
Clinical Officer		10.25	10.29	6.89	8.34
Pharmacist		10.20	10.23	6.89	8.20
Nurse		9.17	9.21	6.12	8.34
Counselor		1.08	0.94	0.82	1.54
Nutritionist		0.20	0.18	0.05	0.07
Doctor		0.07	0.06	0.18	0.23
Dentist		0.04	0.05	0.00	0.00
Ophthalmologist		0.01	0.00	0.00	0.00
Radiologist		0.00	0.00	0.00	0.01

The number of clinic visits in year 2 were likely fewer than in year 1 because the first visit in the second year might not be scheduled until one or two months into the new year. In addition, patients generally are provided with longer prescriptions when stable on ART (which is likely the case for patients retained at year 1).

### Unit costs

Unit costs for the three categories of resources—ARV medications, laboratory tests, and staff encounters—are reported in Tables 5–7. In Table 5, the standard first-line single pill triple combinations, AZT/D4T + 3TC + NVP, cost $0.16 per day (about $57 per year) in 2012, while TDF/3TC/EFV was substantially higher at $0.47 per day ($172 per year). ARV medication costs have typically fallen over time after introduction into national first line regimens. For example, the Clinton Health Access Initiative (CHAI) reference price for TDF/3TC/EFV in 2016 was down to $0.27 per day (a 50% nominal reduction from 2012).[29]

**Table 5:** Cost per day for ARV medications ^a^.

ARV medication	Pills per day	USD 2012 Daily Cost	USD 2016 Daily Cost
AZT/3TC/NVP (300/150/200 mg)	2	0.157	0.273
D4T/3TC/NVP (30/150/200 mg)	2	0.157	0.273
TDF/3TC/EFV (300/300/600 mg)	1	0.473	0.273
EFV (600 mg)	1	0.141	0.113
AZT/3TC (300/150 mg)	2	0.267	0.220
NVP (Nevirapine—200 mg)	2	0.084	0.083
TDF/3TC (300/300 mg)	1	0.217	0.150
D4T/3TC (30/150 mg)	2	0.105	0.150
LPV/r (Lopinavir/ritonavir—200/50 mg)^a^	4	0.667	0.667
AZT (Zidovudine—300 mg)	2	0.231	0.208
ABC/3TC (300/150 mg)	2	0.458	0.458
3TC (Lamivudine—150 mg)	2	0.078	0.075

^a^The 2012 unit cost for LPV/r and ABC/3TC could not be established because no purchases were included in the invoices reviewed, so the 2016 reference price was used in the costing analysis. Almost no days of ABC/3TC and very few days of LPV/r were prescribed during 2012 (see Table 2).

**Table 6:** Laboratory unit cost (fee) per test.

Laboratory tests charge	2012 USD	2016 USD
Full hemogram	11.76	7.04
CD4 Count Assay	25.65	20.77
Alanine Aminotransferase (ALT)	7.06	5.60
Creatinine	1.76	NC^a^
Aspartate Aminotransferase (AST)	7.06	5.61
Venereal Disease Research Laboratory test (VDRL)	1.41	NC
HIV Viral Load	84.12	11.00
Electrolytes	5.29	NC
Pregnancy	1.76	NC
Sputum Acid Fast Bacilli	4.71	NC
Chest X-ray	2.94	NC
Blood Smears (BS) Malaria	0.59	NC
Erythrocyte Sedimentation Rate (ESR)	2.94	NC
Hepatitis	2.35	NC
Cervical	7.06	NC
Stool for ova, cysts and parasite (OCP)	0.47	NC
Widal	1.41	NC
Glucose	1.41	NC
Histology	8.24	NC
Pelvic	2.94	NC
Bone Marrow (BM) Aspiration	0.00	NC
Urinalysis	0.94	NC
XRAY	2.94	NC
Prothrombin time (PT) test (INR)	17.65	NC
CT Scan	17.65	NC
Ultrasound	7.06	NC
Resistance	211.76	NC

^a^ NC = no change.

**Table 7:** Unit costs per staff encounter (USD 2012).

	MCH	ART clinic
Staff Type	USD 2012	USD 2012
Doctor	9.28	9.28
Clinical Officer	2.75	2.65
Nurse	5.05	2.38
Counselor	2.34	1.53
Pharmacist	1.93	1.93
Dentist	9.28	9.28
Nutritionist	2.34	1.53
Opthalmologist	9.28	9.28
Radiologist	9.28	9.28

Laboratory fees for tests are reported in Table 6 for 2012 and 2016. In 2012, among the tests actually ordered, viral load testing ($84) had the highest unit cost, but relatively few were ordered (more in the ART clinic group, but rare in the MCH clinic group). CD4 tests were commonly performed with a unit cost of $24.

As reported in Table 7, the unit cost per encounter with a clinical officer, nurse, and pharmacists varied slightly between the two clinics. The ART clinic has more staff as well as more patients than the MCH clinic (HIV-infected patients). Due to varying staff type to patient ratios, the cost per patient encounter in the MCH clinic is about the same for a clinical officer, somewhat higher for a counselor, and higher for a nurse. For other more specialized care (e.g. a doctor), the patients at both clinics see the same providers so unit costs are identical.

### Incremental costs

The average incremental costs (2012) was $216 (median $202) for the MCH clinic group and $288 (median $228) for the ART clinic group. This average includes patients not retained at year 1 as well as those retained at year 1. Not surprisingly, patients not retained incurred costs below the average for their cohort ($124 for the MCH clinic group and $223 for the ART clinic group).

For the subset of patients retained at one year on ART and two years on ART, Table 8 shows the average (and median) incremental costs of outpatient care per year. In addition, Table 8 also shows the costs for each major input category (drugs, laboratory tests, and staff encounters). For the first year on ART, incremental costs are skewed so that the average cost per patient for the MCH clinic group ($234) was somewhat higher than the median of $215. For the ART clinic group, the average cost of $292 was substantially higher than the median of $227.

**Table 8:** Annual average incremental costs (2012 USD) for patients retained in care^a^.

	Year 1	Year 2	Year 1	Year 2
	MCH clinic	MCH clinic	ART clinic	ART clinic
	n = 66	n = 57	n = 74	n = 62
Annual incremental costs per patient	234: 215, 253 (215: 186, 282)	215: 194, 236 (203: 154, 250)	292: 256, 329 (227: 178, 357)	213: 191, 236 (209: 157, 258)
Of which:				
ARVs	66: 56, 76 (61: 54, 63)	68: 54, 81 (54: 47, 61)	91: 81, 102 (73: 59, 107)	80: 70, 90 (61: 57, 96)
Laboratory tests	69: 60, 78 (55: 54, 107)	80: 67, 93 (57: 54, 109)	138: 107, 169 (96: 54, 161)	96: 79, 113 (94: 54, 132)
Staff encounters	99: 92, 106 (98: 78, 119)	67: 61, 73 (60: 49, 85)	63: 59, 67 (58: 50, 70)	38: 34, 41 (35: 28, 42)

^a^In each cell, row 1 contains the average cost and 95% confidence interval. Row 2 contains the median and interquartile range.

Cost fell somewhat during the second year on ART for the MCH clinic group, from $234 to $215 and costs fell substantially in year two for the ART clinic group (from $292 to $213). The reduction in average costs per year across the two years are explained by fewer staff encounters, slightly fewer laboratory tests, and fewer ARV medications prescribed (as explained above).

The difference in ARV medication costs between the two study groups is largely explained by more use of TDF and EVF in the ART clinic group as well as more days of ARVs prescribed. The difference in laboratory costs between the two groups is largely explained by somewhat more viral load testing in the ART clinic group. The difference in staff encounter costs is largely explained by fewer clinic visits for the ART clinic group.

Although all patients included in the results reported in Table 8 were retained in care, substantial variation still exists across patients, as would be expected based on the days without ARVs reported in Table 1. From Table 8, while the average cost during year one for the MCH clinic group was $234 (median $215), 25% of patients had costs above $282 and below $186

For both clinics, a patient who received the least expensive triple combination therapy along with relatively few labs and clinic visits would have had average costs of at least $175 per year. Thus, very “low cost” patients for both clinics are low cost because they had few clinic visits and a substantial number of days without ARVs during their first year on ART (although defined as retained in care).

### Modeled incremental costs

Table 9 replicates the information on costs of care over the first and second year ART from Table 8 but with: (1) updated 2016 unit costs for drugs and viral load monitoring; (2) TDF+3TC+EFV used as the main triple drug combination according to newer Kenya treatment guidelines [5]; (3) CD4 monitoring replaced by viral load monitoring according to Kenya guidelines [5], and (4) staff encounter costs in USD are held constant.

**Table 9:** Annual average incremental costs (2016 USD) for patients retained in care^a^.

	Year 1	Year 2	Year 1	Year 2
	MCH clinic	MCH clinic	ART clinic	ART clinic
	n = 66	n = 57	n = 74	n = 62
Annual incremental costs per patient (2016 USD)	238: 222, 255 (232: 206, 278)	205: 190, 219 (201: 172, 232)	227: 212, 242 (211: 186, 255)	176: 164, 187 (174: 152, 201)
Of which:				
ARV medications	99: 90, 108 (102: 88, 110)	93: 83, 103 (90: 74, 98)	106: 102, 111 (104: 98, 118)	94: 89, 99 (98: 86, 106)
Laboratory tests	41: 35, 46 (32: 31, 62)	44: 38, 51 (35: 31, 63)	58: 48, 68 (51: 31, 72)	45: 38, 52 (40: 31, 64)
Staff encounters	99: 92, 106 (98: 78, 119)	67: 61, 73 (60: 49, 85)	63: 59, 67 (58: 50, 70)	38: 34, 41 (35: 28, 42)

^a^In each cell, row 1 contains the average cost and 95% confidence interval. Row 2 contains the median and interquartile range.

The unit cost of TDF+3TC+EFV fell substantially over this period, from $0.47 per day in 2012 to $0.27 per day in 2016.[29] The laboratory fee for viral load monitoring also fell from $84 in 2012 to $11 as of the end of 2016.

For the MCH group, the modeled costs for 2016 remained similar to the estimated costs for 2012. The reduction in laboratory unit costs were offset with higher ARV unit costs. For the ART clinic group, costs in 2016 over the first year on ART are modeled at $227, which is down substantially from $292 in 2012 due to the substantial reduction in laboratory costs. In both clinics, costs in year 2 are lower than year 1 ($205 in the MCH clinic and $176 in the ART clinic using 2016 costs) due to fewer clinic visits, longer drug prescriptions, and fewer laboratory tests in year 2.

## Discussion

The primary objective of this paper was to estimate the annual incremental cost of providing ART within an MCH clinic for adult women initiated on ART during pregnancy over the first one and two years on treatment. For perspective, these costs are also compared to costs over the same follow-up period for a cohort of adult, non-pregnant women who initiated ART at the general ART clinic at the same hospital compound.

Two main implications of this analysis for resource use and patient retention outcomes are noted here. First, the average incremental costs for the first year on ART for women initiated during pregnancy and retained at one year at the MCH clinic was $234 (in USD 2012). For non-pregnant women initiated at the general ART clinic and retained at one year, the average incremental costs was $292 (in USD 2012). The difference was largely driven by more costly drug regimens and slightly more laboratory tests in the ART clinic group, even though patients on ART at the MCH clinic made more clinic visits. As reported in Table 8, averages mask the substantial variation in costs at the patient level within the same clinic.

And second, patients retained in care at year one were nevertheless often late for their next visit, where late is defined as having run out of ARVs before making their next visit. In the MCH clinic group for example, while 83.5% of patients were retained after 1 year on ART, over 25% of these patients were without ARVs for at least 68 days (and 54 days for the ART clinic group).

This analysis has important limitations. First, the analysis focused on pregnant women who initiated antiretroviral therapy at the study MCH clinic during a time when eligibility was based on a CD4 count threshold (or WHO stage). Due to record systems at the time, it was not possible to document the proportions of pregnant women newly diagnosed with HIV who were assessed for treatment eligibility, found eligible, offered to initiate ART, and actually initiated ART. Thus, retention reported in Table 1 is only for the subset who actually initiated ART.

Second, due to record systems especially prior to 2012, it was not possible to link data for the MCH clinic group to their infants data (date of birth, birth outcome, HIV testing dates and results, etc.). Thus, it was not possible to evaluate costs and retention outcomes along the PMTCT cascade of care.

In addition, since a delivery date is not available, it is not possible to determine how many visits occurred during the antenatal period. All patients in the MCH clinic group received HIV-related care along with standard antenatal care at clinic visits before delivery. Thus, the costs per patient for HIV care conceputally could exclude any standard antenatal services jointly provided during the antenatal period. For the resources included in Table 8, the main result would be to reduce the costs somewhat of encounters with clinic staff.

Third, the sample available was smaller than originally expected. HIV prevalence among women presenting for a first ANC visit was falling during this time period, and not all HIV-infected pregnant women were eligible for treatment due to the CD4 eligibility threshold. In addition, not all eligible women initiated treatment, and a substantial number of HIV-infected pregnant women were presenting for antenatal care already on ART. And last, patients transferred out of the study site for care and treatment after their HIV diagnosis.

Fourth, non-ARV medications were excluded from incremental costs. Across all patients, antibiotics (Cotrim, Dapsone) and multivitamins were most commonly prescribed along with antihistamines (Cetrizine and Acetaminophen). While a number of different medications were prescribed across all patients, any one patient received few prescriptions beyond the four drugs mentioned above. Thus, the results presented in Table 8 are best interpreted as the incremental cost of HIV care and treatment rather than all medical care and treatment.

Given the focus on incremental costs, program-level costs above the clinic level and fixed costs at each clinic are excluded from the analysis for two main reasons. First, fixed costs are not directly related to patient care, so in general do not change as additional (incremental) patients are treated at a clinic. For perspective, as part of another study, fixed costs at the ART clinic included in this study were estimate at $48 per year per patient in 2012.[30] And second, economic theory provides no conceptually correct way to allocate fixed costs to patients (HIV positive or negative in the MCH, those on ART and those pre-ART at the ART clinic, those completing many visits, those having fewer visits, etc.). Multiple approaches have been used in the existing literature, including fixed costs per patient per year, fixed costs per patient per month they are “in care”, and fixed costs per visit, sometimes pro-rated differently for pre-ART and ART patients. None, however, are grounded in economic theory.

Despite these limitations, this analysis highlights three important issues for future evaluation of service delivery for pregnant and post-partum women and their infants. First, the details of visit dates and drug provision is crucial of understanding adherence to medications during and after delivery. Existing literature does not adequately compare visit schedules to drug prescriptions. For example, the typical definition of “loss to follow-up” is 90 days late for the last scheduled visit, which allows for 89 days of missed drugs after the last visit to remain defined as retained.[27] In the first year on ART, this could easily represent 30% of the year. In the future, an uninterrupted supply of drugs during key time periods (from first ANC visit to delivery; from delivery to 6, 12, 18 months; etc.) needs to be better documented to identify where/when clinics need to focus on improving adherence to clinic visit schedules.

Second, given the consolidation of care and treatment guidelines for adults (e.g., essentially the same first-line regimen and laboratory testing for pregnant and non-pregnant adults initiating treatment), the incremental ARV medication costs for pregnant women are going to be very similar to adults in general (as long as prescription lengths are similar). Laboratory costs are also likely to be somewhat higher for pregnant and post-partum women through breast feeding because of somewhat more frequent viral load testing, which could result in faster switching to second-line regimens if needed.[7]

And third, only further modest reductions in incremental costs can be obtained through longer prescriptions and as a result fewer patients visits. For the ART clinic group for 2016 from Table 9, for example, staff/visit costs were 27% of total incremental costs (with about 8 visits during year 1). A large reduction in visits (e.g., a 66% reduction in visits) would reduce total incremental costs by 18%.

Successful implementation of the PMTCT services requires that: (1) pregnant women with HIV initiate life-long ART with minimal delay after their first ANC visit (and women already on ART should continue on ART); (2) they continue on treatment through delivery; (3) they continue on treatment after delivery and the cessation of breastfeeding (and continuing for life for their health); and (4) their child completes HIV testing at 6, 24, 48, and 72 weeks so that they are known to be uninfected after the cessation of breast feeding. Further evaluation research remains needed to assess outcomes along the PMTCT cascade of care during the new era of treatment for all, to assess costs of care, and to evaluate cost effectiveness of additional strategies to improve implementation of PMTCT services during pregnancy for the mother and after delivery for the mother and child.
